# New karyotype records for the genus *Proechimys* (Rodentia: Echimyidae) from Brazilian Amazonia

**DOI:** 10.1590/1678-4685-GMB-2019-0093

**Published:** 2020-06-01

**Authors:** Eduardo Schmidt Eler, Carlos Eduardo Faresin e Silva, Maria Nazareth Ferreira da Silva, Eliana Feldberg

**Affiliations:** 1 Universidade Anhembi Morumbi, Laureate International Universities, Escola de Ciências da Saúde, São José dos Campos, SP, Brazil; 2 Instituto Nacional de Pesquisas da Amazônia, Coordenação em Biodiversidade, Laboratório de Genética Animal, Manaus, AM, Brazil; 3 Instituto Nacional de Pesquisas da Amazônia, Coordenação em Biodiversidade, Coleção de Mamíferos, Manaus, AM, Brazil

**Keywords:** Spiny rats, rainforest, FISH, 18S rDNA, chromosome rearrangements

## Abstract

We present new karyotype records for six *Proechimys* species from the Brazilian Amazon. *P. echinothrix* from the region of Purus River had 2n = 32 chromosomes and a FN = 58, while *P. cuvieri* from the region of the Japurá River presented 2n = 28 and FN = 46. All individuals presented hybridization with an 18S rDNA probe in a single chromosome pair, with the exception of *P. cuvieri* from the Japurá region, which presented a third signal in one of the homologs of pair 1. No ITS were found in any of the individuals. Our data supports the hypothesis that the *P. cuvieri* population from the Japurá Basin and *P. echinothrix* from the lower Purus are new taxonomic entities. Our data expand the geographic distribution of the cytotype (2n = 40, FN = 54) described for *P. gardneri* from the Madeira River, and the cytotype (2n = 46, FN = 50), described for *P. guyannensis*, as well as the recently-described cytotype of *P. goeldii* (2n = 16, FN = 14). No clear pattern of chromosomal evolution has yet been defined in *Proechimys*, despite the considerable karyotypic diversity of the genus.

## Introduction

The Neotropical rodents of the family Echimyidae are considered the most diverse of the infraorder Hystricognathi, not only in terms of their taxonomy, but also their ecology, morphology, and adaptations (Araújo *et al.*, 2014). The Echimyidae, which includes approximately 90 species in 19 genera (Woods and Kilpatrick, 2005; Patton and Leite, 2015), is a prime example of major adaptive radiations in the Hystricognathi (Fabre *et al.*, 2012). The echimyid genera include *Proechimys*, which is the most species-diverse, with 22 species (*sensu* Patton and Leite, 2015), distributed primarily in Amazonia (da Silva *et al.*, 2001; Iack-Ximenes *et al.*, 2005; Patton and Leite, 2015). However, *Proechimys* is taxonomically problematic, due to the phenotypic similarities among its species and its considerable intraspecific variation. The cytogenetics of *Proechimys* is also complex, with diploid numbers ranging from 14 to 62 chromosomes and at least 62 known karyotypes (Eler *et al.*, 2012; Amaral *et al.*, 2013).

A number of studies have shown that non-codifying repetitive DNA sequences play a fundamental role in cell maintenance, and are involved in regulatory mechanisms that determine gene expression. These sequences may result in phenotypic changes, as well as being involved in the speciation process through the evolution of the host genome (Kazazian, 2004; Martins, 2007; Vitte *et al.*, 2014; Zeigler, 2014). Given this, the physical-chromosomal mapping of repetitive sequences may provide important insights into the structure of the genome and generate chromosomal markers, which may be extremely valuable for evolutionary studies, including the identification of specific chromosomes and rearrangements, and in particular the identification of sex chromosomes (Martins, 2007).

In the family Echimiydae, the mapping of repetitive DNA sequences is limited to the location of the 45S rDNA and telomeric sequences in six species: *Phyllomys lamarum* and *Phyllomys* sp. from northern Minas Gerais, Brazil (Araújo *et al.*, 2014); and *Proechimys guyannensis, Proechimys cuvieri* (Silva *et al.*, 2012), *Proechimys longicaudatus* (Amaral *et al.*, 2013), and two cytotypes of *P. goeldii* (Rodrigues da Costa *et al.*, 2016), collected in Brazilian Amazonia.

Given the persistent taxonomic uncertainties in *Proechimys*, the existence of the same diploid number in different species, different diploid numbers in the same species, the occurrence of sympatry in many species, and the relatively recent diversification of the genus (which together make it an excellent model for evolutionary studies in the echimyids), the objective of the present study was to provide new karyotype data for the genus. The findings include the location of the 18S rDNA sequences and the telomeric DNA in six *Proechimys* species, based on individuals collected from different sites located throughout the Brazilian Amazon region. These data were compiled in an attempt to decipher the chromosomal mechanisms involved in the evolution of the genus.

## Material and Methods

**Figure 1:**
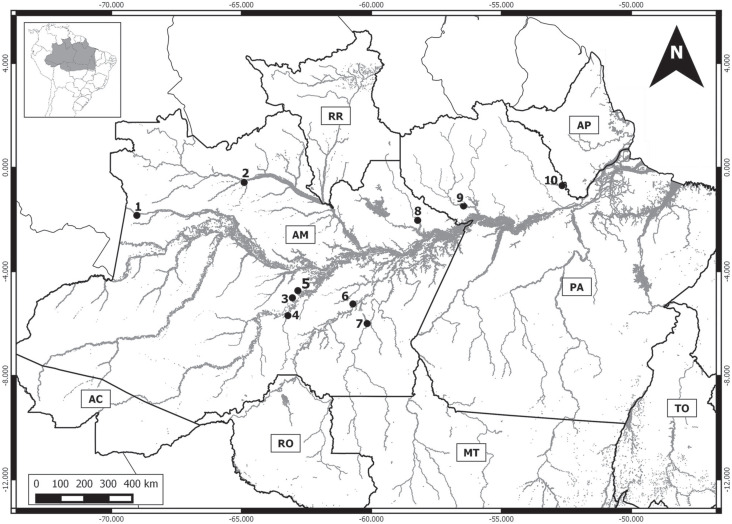
Map of the Brazilian Amazon basin, indicating the collection locations of *Proechimys* specimens. 1 - left bank of up Japurá River (1.84341666667°S, −69.0264722222°W); 2 - Santa Isabel do Rio Negro, Negro River (0.57725000000°S, 64.8976944444°W); 3 - Extractive Reserve of Canutama, Purus River (6.5784111000°S, 64.5723388900°W); 4 - lower Jacinto River, right bank of Purus River (6.8300640000°S; 64.2819020000°W); 5 - Rebio Abufari, left bank of Purus River (4.97616666667°S; 62.9774722222°W); 6 – Left bank of Madeira River (4.97616666667ºS, 62.9774722222ºW); 7 - lower Aripuanã River (6.00000000000° S, 60.1666666667°W); 8 - Jatapú River (2.017940° S, 58.203228° W); 9 - Flona Saracá-Taquera e Rebio Trombetas, Trombetas River (1.48163888889° S, 56.4573333333° W); 10 - Jari River valley (0.7000000000° S, 52.6666666667° W).

In the present cytogenetic study, we analyzed 56 *Proechimys* individuals representing six species (Table 1), collected at 10 localities in the Brazilian Amazon basin (Figure 1), which were deposited in the mammal collection of the National Institute of Amazonian Research (INPA) in Manaus, northern Brazil. The collection of the individuals for scientific research was authorized by licenses 02005.000642/03-11 (IBAMA/MMA), 02000.002336/ 2003-93 (IBAMA/MMA), 02005.002672/04 (IBAMA/MMA), 37585-5 (SISBIO/MMA), 37592-4 (SISBIO/MMA), 10985 (SISBIO/MMA), and the research was approved by the INPA Committee on the Ethical Use of Animals in Research (protocol number 02/2013).

**Table 1:** Species, number of specimens by sex, origin, and voucher specimens analyzed in the present study.

Species	Number of individuals		2n/FN	Locality (and its number in Figure 1)	Vouchers
	Male	Female			
*P. gardneri*	2	6	40/50	Aubufari Biological Reserve, Amazonas (5); Left Bank of Madeira river, Amazonas (6)	INPA4796, INPA5376, INPA5383, INPA5390, INPA5391, INPA5395, INPA5396, SIS881
*P. guyannensis*	1	4	46/50	Saracá-Taquera National Florest and Trombetas Biological Reserve, Pará (9)	SIS1488, SIS1519, SIS1603, SIS1620, SIS1677
*P. guyannensis*	6	3	38/52	Santa Isabel do Rio Negro, Amazonas (2); Jari river valley, Pará e Amapá (10)	INPA5044, INPA5045, INPA5052, INPA5053, INPA5054, INPA5229, SIS423, SIS553, SIS607
*P. goeldi*		1	16/14	down Jacinto River, right bank of Purus river, Amazonas (4)	SIS1073
*P. echinothrix (new citotype)*	2	2	32/58	Extractive Reserve of Canutama, Purus river, Amazonas (3); down Jacinto river, right bank of Purus river, Amazonas (4)	SIS1060, SIS1084, CAN23, CAN49
*P. cuvieri (new citotype)*	1	3	28/46	lefft bank of down Japurá river, Amazonas (1)	SIS1978, SIS1979, SIS1849, SIS1909
*P. cuvieri*	9	7	28/46	down Aripuanã river, Amazonas (7); Jatapú River, Amazonas (8);, Saracá-Taquera National Florest and Trombetas Biological Reserve, Para (9), Jari river valley, Pará (10)	INPA5050, SIS74, SIS164, SIS1634, SIS1646, SIS1653, SIS1666, SIS1670, SIS1679, SIS1689, SIS1724, SIS1773, EE172, EE238, EE251, EE252
*P. longicaudatus*	7	2	28/46	Left Bank of Madeira river, Amazonas (6)	INPA4749, INPA4754, INPA4757, INPA4762, INPA4764, INPA4789, INPA5401, INPA5410, INPA5414

* Vouchers being processed are identified by field number. Vouchers already processed are identified by a registry number.

Cell suspensions were obtained from bone marrow using the “air-drying” method of Ford and Harmerton (1956) with modifications. Cell division was blocked *in vivo* using colchicine at a concentration of 0.0125% (defined by the best distension of the chromosomes), at a proportion of 1 mL per 100 g of body weight, for 20 min. The bone marrow was removed from the femur using jets of hypotonic solution (0.075 M KCl) and maintained in this solution for 30 min at 37 ºC. The samples were then washed three times (10 min each time) in Carnoy fixative after pre-fixation. The cell suspensions were dripped onto glass slides and stained with 5% Giemsa for 10 min. The constitutive heterochromatin was stained by C-banding according to Sumner (1972). The Nucleolus Organizer Region (NOR) was located using the procedure described by Howell and Black (1980).

Repetitive regions of the 18S rDNA and telomeres were mapped by fluorescence *in situ* hybridization (FISH) (Pinkel *et al.*, 1986) with adaptations, at a stringency of 77%. The probes were obtained using standard primers for mammals. For the 18S rDNA probe, the primers were F 5'-CCG CTT TGG TGA CTC TTG AT-3' and R 5'-CCG AGG ACC TCA CTA AAC CA-3' (Gross *et al.*, 2010), while for the telomeric region(Ijdo *et al.*, 1991), they were (TTAGGG)_5_ and(CCCTAA)_5_. The PCR products of the 18S rDNA gene and the telomeric sequence were marked by nick translation with digoxigenin-11-dUTP (Dig-Nick Translation mix; Roche), following the manufacturer's instructions. Hybridization signals were detected using anti-digoxigenin-rhodamine (Roche Applied Science). The chromosomes were then counterstained with DAPI and analyzed under an Olympus BX51 epifluorescence microscope. The chromosomes were paired considering the morphology in decreasing order of size, with the chromosomes being classified as metacentric (m), submetacentric (sm), subtelocentric (st) or acrocentric (a), based on the ratio of the chromosome arms and the position of the centromere (see Patton, 1967). The fundamental number was determined considering only the autosomal arms (FNa).

## Results

We extended the known geographic distributions of *P. gardneri, P. guyannensis* and *P. echinothrix* by surveying areas not previously sampled for these species. We also recorded new data in the chromosome complements of *P. cuvieri, P. goeldii*, and *P. echinothrix*. Data on the localization of 18S rDNA and telomeric sequences are presented for all studied species.

The data on the macrostructure of the karyotypes (2n, FNa, NOR, C- and G-banding) of *P. gardneri* (2n = 40, Fna = 54) from the region of the middle Madeira River (locality 6, Figure 1), *P. longicaudatus* (2n = 28, FNa = 46) from the Aripuanã River (locality 7), and *P. guyannensis* (2n = 38, FNa = 52) from the region of Santa Izabel (locality 2), on the Negro River, and Vale do Jari (locality 10) were presented by Eler *et al.* (2012). In the present study, we added data on the 18S rDNA and telomeric markers (by FISH) for the species and localities.

The *P. gardneri* individuals collected from the Abufari Biological Reserve, on the right margin of the Purus River (locality 5) had 2n = 40 (12m + 4sm + 22a + XX/XY) and FNa = 54, with acrocentric X and Y chromosomes of medium and small size, respectively (data not shown). The simple NOR was located interstitially on the long arm of the second submetacentric pair (8). Blocks of heterochromatin were found on four metacentric pairs (3–6), on all the acrocentric chromosomes, and on the X chromosome.

The *P. guyannensis* individuals from the Saracá-Taquera National Forest (locality 9, right margin of the Trombetas River) and the Trombetas Biological Reserve (locality 9, left margin) had a 2n = 46 (4m + 2sm + 38a + XX/XY) and FNa = 50, with acrocentric pairs 4, 5, and 6 being significantly larger than all the others of the complement (data not shown). The X and Y chromosomes are acrocentric, and the Y chromosome is approximately half the size of the X. The NOR is simple, located interstitially on the long arm of pair 3 (sm). Heterochromatin blocks were observed in the centromeric region of 10 autosomal pairs and the X chromosome, while the Y chromosome is totally heterochromatic.

**Figure 2:**
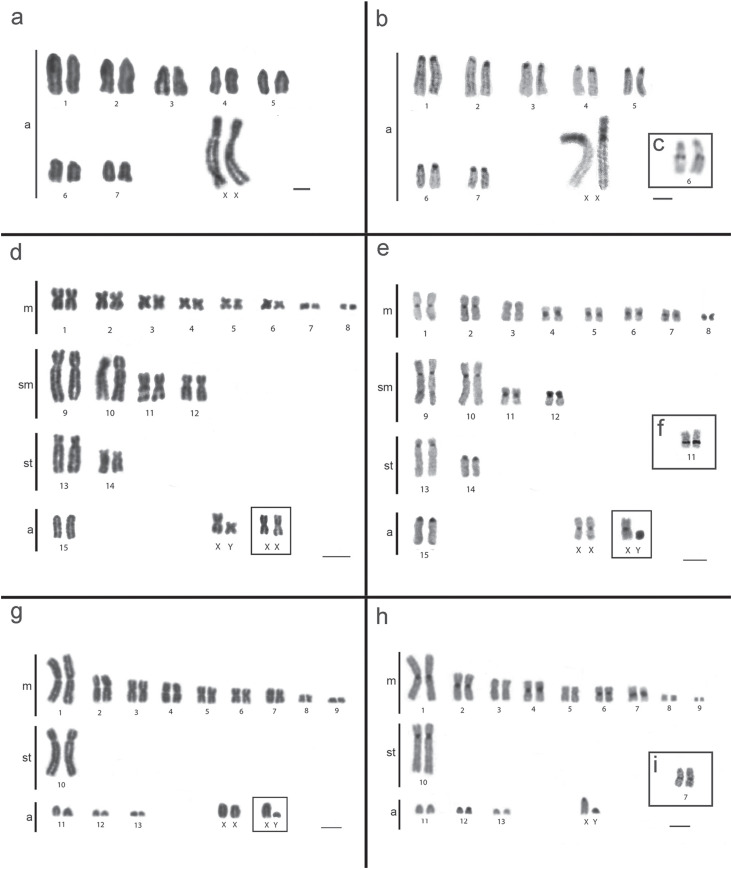
Karyotypes of *Proechimys goeldii* (2n = 16): (a) conventional staining, (b) C-banding, (c) NOR. Karyotypes of *Proechimys echinothrix* (2n=32): (d) conventional staining, (e) C-banding, (f) NOR. Karyotypes of *Proechimys cuvieri* (2n=28): (g) conventional staining, (h) C-banding, (i) NOR. Bars = 10 μm.

The *P. goeldii* individual, from the Jacinto Stream, on the right margin of the Purus River (locality 4) had 2n = 16 (14a + XX/XY) and FNa = 14. The X chromosome is the largest submetacentric (Figure 2a). As the only specimen of this species is a female, we have no data on the Y chromosome. The blocks of constitutive heterochromatin are located in the centromeric region of all the autosomal chromosomes, while in the X chromosome, in addition to the centromeric region, the short arms are completely heterochromatic (Figure 2b). The NOR is simple, located interstitially on the long arms of pair 6 (Figure 2c).

In *P. echinothrix* from the Canutama Extractive Reserve (left margin of the Purus River; locality 3) and the Jacinto stream (right margin of the Purus river; locality 4), we found 2n = 32 (16m + 8sm + 4st + 2a + XX/XY) and FNa = 58. The sex chromosomes are metacentric, and the X is practically twice the size of the Y (Figure 2d). Pairs 9 and 10 (sm), and 13 (st) are significantly larger than all the others of the complement. The blocks of constitutive heterochromatin are located in the centromeric region of all the autosomes and the X chromosome. The short arm of pair 12 is completely heterochromatic (Figure 2e). The simple NOR is located interstitially on the long arms of pair 11 (sm), coinciding with the secondary constriction (Figure 2f).

The *P. cuvieri* individuals collected at the Taboca community, on the Japurá (locality 1), had 2n = 28 (18m + 2st + 6a + XX/XY) and FNa = 46. The X and Y chromosomes are acrocentric, and the X is significantly larger than the Y chromosome (Figure 2g). The constitutive heterochromatin is located in pericentromeric blocks in chromosome pairs 1, 2, 4, 5, 6 (m), 10 (st), and 12 (a), and also in the centromere of the X chromosome. The Y chromosome is completely heterochromatic (Figure 2h). The NOR is simple, located in the interstitial region of the long arms of pair 7 (Figure 2i).

The *P. cuvieri* individuals collected from the region of the Jatapu (locality 8), where the species occurs in sympatry with *P. guyannesis*, and from locality 9 have 2n = 28 (14m + 4sm + 2st + 6a + XX/XY) and FNa = 46 (data not shown). The X and Y chromosomes are acrocentric, and the X is larger than the Y. The NOR is simple, located in the interstitial region of the long arms of pair 7. The constitutive heterochromatin is located in pericentromeric blocks in pairs 5, 6, 7, 9, 11, 12, and 13, and in the X chromosome.

**Figure 3:**
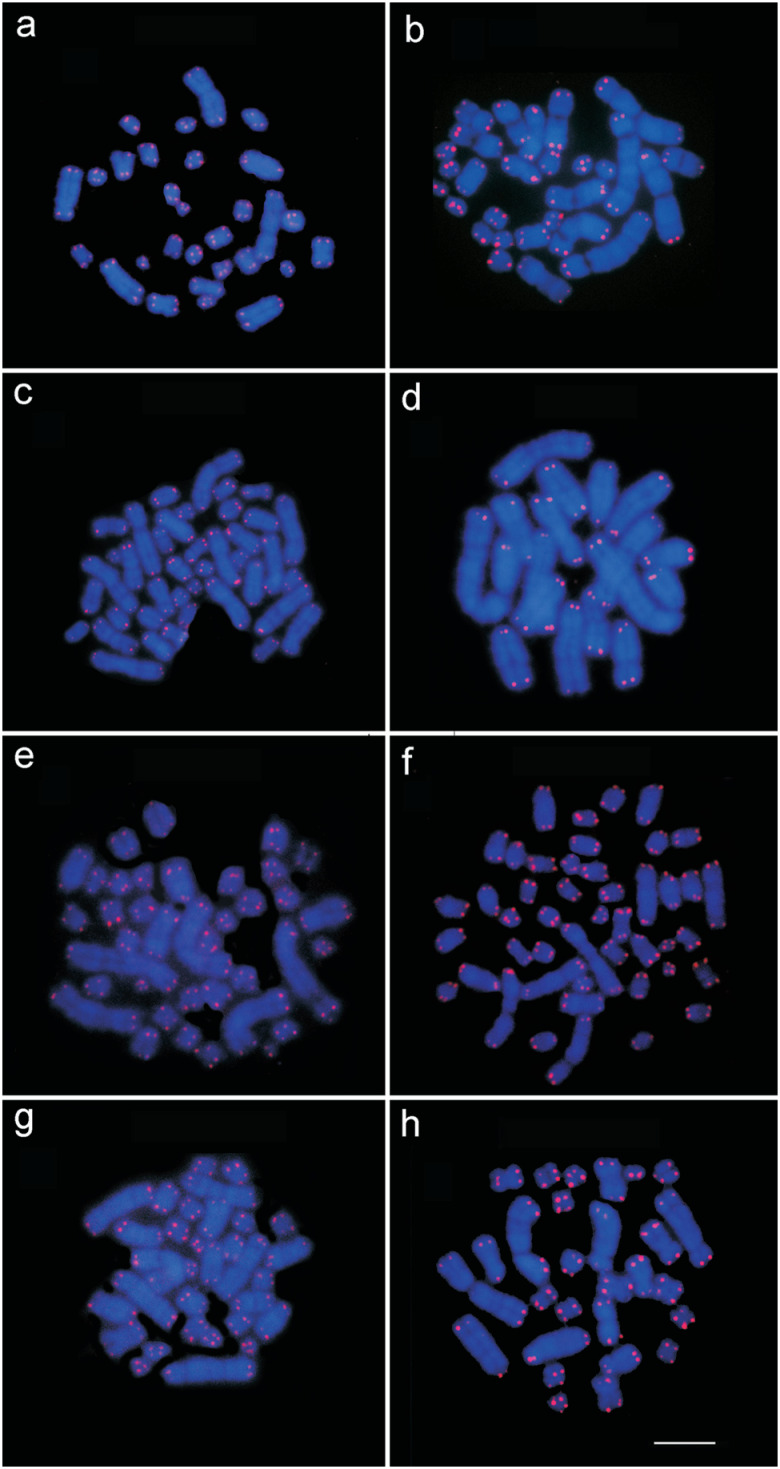
Telomeric marks in *Proechimys cuvieri* (a) from localities 7, 8 and 9; (b) *P. cuvieri* from locality 1; (c) *P. gardneri* from localities 5 and 6; (d) *P. goeldii* from locality 4; (e) *P. guyannensis* from localities 2 and 10; (f) *P. guyannensis* from locality 9; (g) *P. echinothrix* from localities 3 and 4; (h) *P. longicaudatus* from locality 6. Bar = 10 μm.

**Figure 4:**
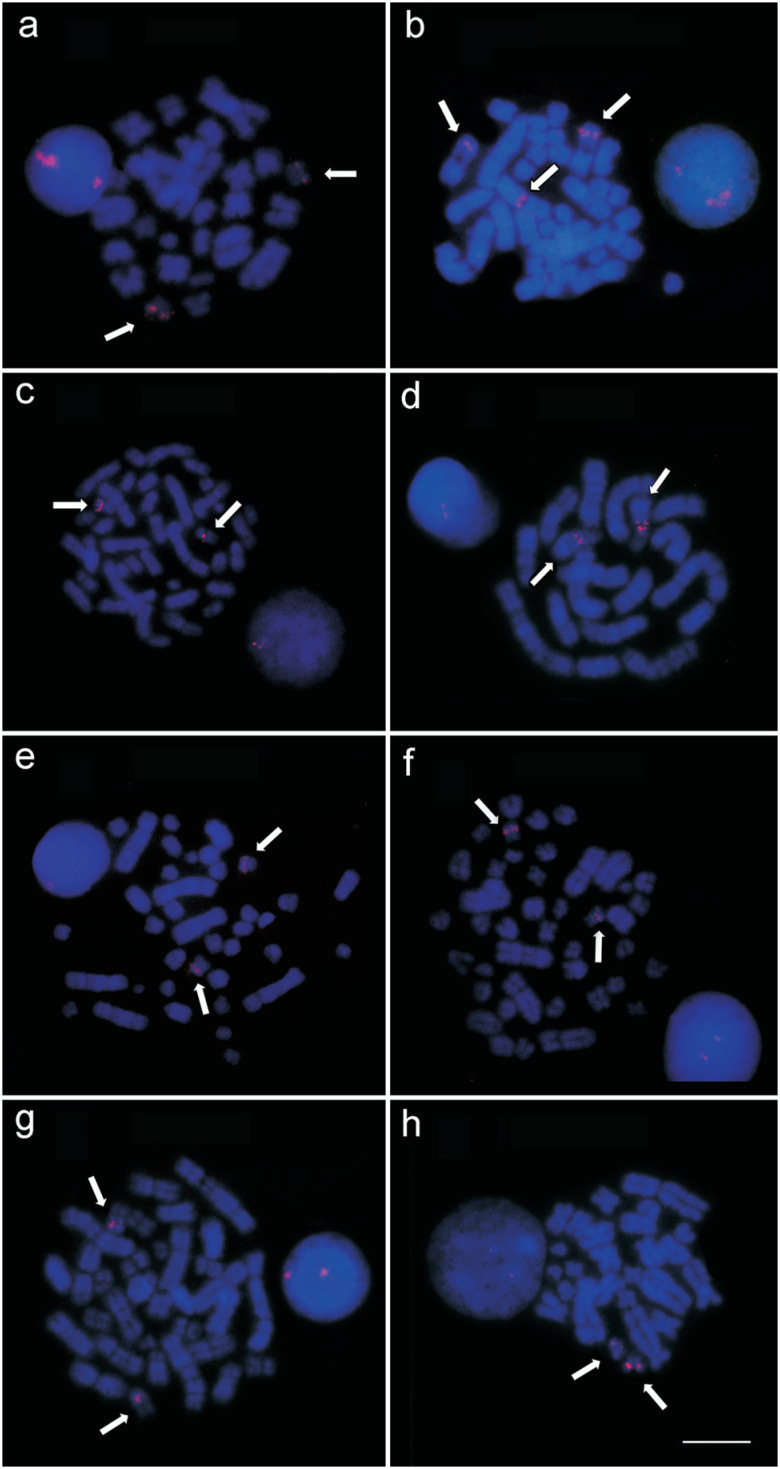
18S DNA labeling (indicated by arrows) (a) in *Proechimys cuvieri* from localities 7, 8 and 9; (b) *P. cuvieri* from locality 1; (c) *P. gardneri* from localities 5 and 6; (d) *P. goeldii* from locality 4; (e) *P. guyannensis* from localities 2 and 10; (f) *P. guyannensis* from locality 9; (g) *P. echinothrix* from localities 3 and 4; (h) *P. longicaudatus* from locality 6. Bar = 10 μm.

Similarly, all the individuals analyzed in the present study hybridized the 18S rDNA probe in a single chromosome pair (the NOR-bearing pair), with the exception of the five *P. cuvieri* individuals from locality 1, which presented a third signal in the pericentromeric region and heterochromatin in one of the homologs of pair 1 in all the cells (Figure 3). All the individuals analyzed here (Table 1) presented telomeric signals in the terminal regions of all the chromosomes, but we found no evidence of the presence of interstitial telomeric sequences (ITSs) in any of the individuals (Figure 4).

## Discussion

The phylogeny of the echimyid genus *Proechimys* is poorly resolved, hampering the understanding of the relationships among its species (Patton *et al.*, 2000). Recent cytogenetic studies have provided increasingly valuable markers for the understanding of the evolution of the genus, given that, while the diploid number does not vary, there are meaningful differences in the chromosome morphology, which are reflected in the fundamental number (FN), and thus in the position of the other markers in the karyotype, as in the location of the nucleolus organizer region (NOR). Cytotaxonomic differences have also been found among species in the sex chromosomes, which have resulted from rearrangements, such as inversions, translocations, and the addition or deletion of heterochromatin. In addition to the fact that the taxonomy of the echimyids is still uncertain, a number of other factors limit the understanding of the true diversity of these rodents, such as the paucity of distributional data and the probable existence of cryptic species. In most cases, in addition, the cytogenetic data are limited to fundamental and diploid numbers (Nagamachi *et al.*, 2015).

**Figure 5:**
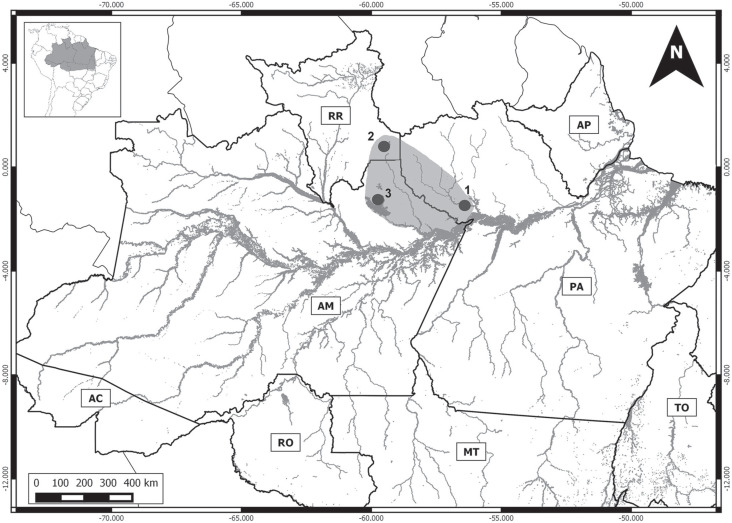
Geographic distribution (dark area) of the 2n = 46 *P. guyannensis* cytotype, showing the collecting localities: 1 – Saracá-Taquera National Forest and Trombetas Biological Reserve, in Pará state (present study); 2 – São João da Baliza, Amapá (Bonvicino *et al.*, 2005); 3 – Balbina hydroelectric reservoir, on the Uatumã River, in Amazonas state (Silva *et al.*, 2012).

In the present study, we have expanded the available chromosomal data for a number of *Proechimys* species, and have added markers for others. In the case of *P. gardneri*, for example, the karyotype recorded from the Abufari Biological Reserve is identical to that recorded in individuals collected on the left margin of the Madeira River (Eler *et al.*, 2012; Figure 5), including the position of the NOR and distribution of the heterochromatin. This allows us to confirm that this cytotype is distributed from the left margin of the Madeira as far west as the left margin of the Purus, and possibly as far as the Juruá River, which would be consistent with the probable distribution of the species, and would imply sympatry with the cytotype described by da Silva (1998) for the Purus-Juruá interfluve.

In the case of the cytotype of *P. guyannensis*, which has 2n = 46, the karyotype observed in the individuals from the Saracá-Taquera National Forest and the Trombetas Biological Reserve (present study) is the same as that recorded in the *P. guyannensis* individuals collected from islands of the Balbina hydroelectric reservoir on the Uatumã River (Silva *et al.*, 2012; Figure 2). The distribution of this cytotype is thus extended approximately 330 km to the east of its previous limit, reaching the margins of the Trombetas River.

Seven cytotypes have been described for the *guyannensis* group (*sensu* Patton, 1987), with diploid numbers of 30, 38, 40, and 46 chromosomes (Machado *et al.*, 2005; Eler *et al.*, 2012; Silva *et al.*, 2012). The 2n = 46 cytotype is found in central Brazilian Amazonia, on the left margin of the Amazon River, in the region between the Uatumã River, in Amazonas state, São João da Baliza, in Roraima, and the lower Trombetas River, in Pará (Figure 5), where it is sympatric with *P. cuvieri*. Given the ample geographic distribution of the species of the *guyannensis* group, and their considerable diversity of cytotypes, we believe that this group may contain a number of different species that have yet to be described formally.

The three known *P. cuvieri* karyotypes (2n = 28 and FN = 46, FN = 48, and FN = 50) include three pairs of chromosomes that are significantly larger than all the others of the complement, together with at least one submetacentric pair (Maia and Langguth, 1993; Patton *et al.*, 2000; Eler *et al.*, 2012; Silva *et al.*, 2012). The individuals from the Japurá River analyzed here (2n = 28, FN = 46) are distinct from all other representatives of this species in having only two pairs (1 and 10) of large chromosomes and no submetacentric chromosomes, with a relatively larger number of acrocentrics, in comparison with the karyotypes described previously. The distribution of the heterochromatin in the individuals analyzed here is similar to that observed in the individuals from the Uatumã River, with signals being found invariably in the centromeric region of most chromosome pairs (including the X chromosome), with a completely heterochromatic Y chromosome. However, the position of the NOR in the complement varied due to the rearrangements, which altered the chromosomal morphology. Clearly, rearrangements of the pericentric inversion type have occurred in this species, given the alterations in the morphology of the chromosomes, even though the diploid number remained constant.

**Figure 6:**
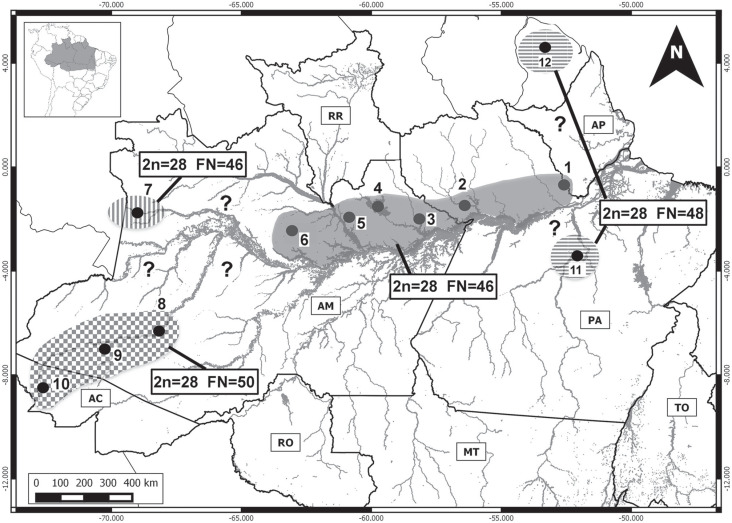
Geographic distribution of the known *P. cuvieri* cytotypes. Dark area: 1 - Vale do rio Jari, in Pará state (Eler *et al.*, 2012); 2 - Saracá-Taquera National Forest and Trombetas Biological Reserve, Pará (present study); 3 - lower Jatapú River, Amazonas (present study); 4 - Balbina hydroelectric reservoir, Uatumã River, Amazonas (Maia and Langguth, 1993; Silva *et al.*, 2012); 5 - Rio Negro State Park, southern sector, Cueiras River, Amazonas (Silva *et al.*, 2012), 6 - Jaú National Park, Amazonas (Patton *et al.*, 2000); Area with vertical hatching (new cytotype): 7- upper Japurá River (present study); Area with cross hatching: 8, 9 and 10 - lower-mid, upper-mid, and headwaters of the Juruá River respectively (Patton *et al.*, 2000); Area with horizontal hatching: 11 - Altamira, Xingu River, Pará (Patton *et al.*, 2000), 12 - La Trinité Mountains, French Guiana (Reig *et al.*, 1979).

Given the karyotypic differences found in *P. cuvieri* from the Japurá basin, we believe that this population may represent a new *Proechimys* species. By contrast, the karyotype of *P. cuvieri* from the Jatapú basin is similar to that recorded for this species from the Balbina reservoir on the Uatumã River (Maia and Langutth, 1993), Manaus and the Cuieiras basin (Silva *et al.*, 2012), the Jaú basin (Patton *et al.*, 2000), and Vale do Jari (Eler *et al.*, 2012), although in this latter case, a small difference was found in the morphology of the sex chromosomes. Up to now, the geographic distribution of this cytotype has been restricted to the north of the Solimões-Amazon channel, from the Jaú National Park eastward virtually as far as the mouth of the Amazon River (Vale do Jari). However, the exact geographic limits are still unclear between the three known *P. cuvieri* cytotypes – 2n = 28 and FN = 46, with two distinct arrangements (Patton *et al.*, 2000; Eler *et al.*, 2012; Silva *et al.*, 2012; present study), 2n = 28 and FN = 48 (Reig *et al.*, 1979; Patton *et al.*, 2000) (Figure 6).

In the case of *P. echinothrix* from the Canutama Extractive Reserve and the lower Jacinto Stream, we recorded a karyotype that is different in a number of aspects from that described by da Silva (1998), i.e., 2n = 32, FN = 60, 20m-sm + 10st + XY, with small acrocentrics. The new cytotype described here for *P. echinothrix* varies principally in the morphology of the sex chromosomes, in addition to the presence of a medium-sized acrocentric pair, and a reduction in the number of subtelocentric chromosomes, from 10 to 4 (Figure 2d). One other diagnostic chromosome marker is the presence of completely heterochromatic short arms in the autosomal pair 12 (Figure 2e), a feature recorded for the first time in the genus *Proechimys*.

**Figure 7:**
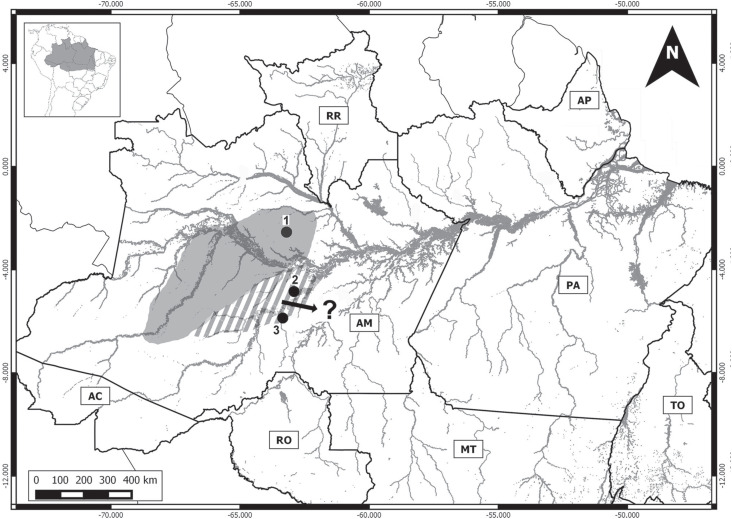
Geographic distribution of *P. echinothrix.* Dark area: known distribution of the species, extended to the region of the Jaú National Park (3) (Patton *et al.*, 2000). Hatched area: proposed extension to the Canutama Extractivist Reserve (2) and the lower Jacinto River (3).

The maintenance of the diploid number in *P. echinothrix*, together with the variation in the fundamental number (even considering potential divergences in the classification of the chromosome morphology) points to the role of pericentric inversions in the rearrangement of these karyotypes. It is interesting to note that the geographic distribution proposed by da Silva (1998) for this species restricts its occurrence to the left margin of the Juruá as far as the east of the upper Urucu basin. In this case, the results of the present study extend this distribution to both margins of the Purus River (Figure 7).

Amaral *et al.* (2013) described a very different *Proechimys* karyotype, in terms of both the diploid number, karyotype formula and multiple sex chromosome (2n = 16/17 e FN = 14), from individuals collected in southern Amazonia, in the Brazilian state of Mato Grosso, which they classified as *P.* cf. *longicaudatus*. The authors also proposed that *P.* cf. *goeldii* from the northern Mato Grosso state, which has the same karyotypic characteristics (studied by Machado *et al.*, 2005) should be reclassified to *P.* cf. *longicaudatus*. Rodrigues da Costa *et al.* (2016) studied two populations of *P*. cf. *longicaudatus* from the western Pará state showing the same karyotypic characteristics found by Amaral *et al.* (2013), and based phylogenetic analyses using the *cyt b* gene, have suggested that this new cytotype (including data from Amaral *et al.*, 2013) represents a distinct species unrelated to *P. longicaudatus* or any representative of the group of *P. goeldii*, diverging from the propositions by Machado *et al.* (2005) and Amaral *et al.* (2013), and remaining with uncertain status in the species groups proposed by Patton (1987). In the present study, we recorded the same cytotype in a female from the lower Purus basin, which almost certainly belongs to this same species. However, unpublished molecular data (MNF Silva, personal communication) link this individual from the Purus River to *P. goeldii*. This extends the distribution of this cytotype (2n = 16, FN = 14) to the left margin of the lower Purus.

The 18S rDNA probe confirmed the presence of a single nucleolar pair in all six species, as shown by the silver staining. As this sequence plays an important role in transcription, the NOR is invariably preserved, despite the structural rearrangements in the autosomal complement of *Proechimys* (which include the NOR-bearing pair, as in the *P. goeldii* individuals analyzed here). This is essential to guarantee the functionality of this sequence in the genome. The NOR-bearing pair is considered to be homeologous, not only among *Proechimys* species, but in the echimyids as a whole (Yonenaga-Yassuda *et al.*, 1985; Eler *et al.*, 2012). We nevertheless recorded a third pericentromeric 18S rDNA signal in *P. cuvieri* from the Jatapú basin (Figure 4b), which coincided with a region of non-active heterochromatin. This signal may represent a fragment of the ribosomal sequence that was left over during rearrangements or transferred from a different region of the genome by transposable elements.

The presence of additional 18S rDNA signals, while uncommon in most vertebrates, is also related to the natural origin and disappearance of repetitive sequences in the genome (Rooney and Ward, 2005). In their study of the variation in the 18S rDNA signal in 19 species of the rodent genus *Mus*, Cazaux *et al.* (2011) verified the association of these sequences with centromeric regions, and suggested that these regions represent a hotspot of chromosomal breakage. However, these authors concluded that the enormous diversity of karyotypes found in *Mus* cannot be accounted for solely by rearrangements, but must also have been determined by other factors, such as epigenetic modifications of the DNA. Here, we present the first evidence of multiple 18S rDNA signals in *Proechimys*, which was not associated systematically with the karyotypic diversity observed in this genus. Even if the region is transcriptionally inactive (not confirmed by the Ag-NOR) and not associated with chromosomal breakage events, it can be considered to be a cytological marker of the *P. cuvieri* population from the region of the Japurá River.

Despite the many rearrangements identified in *Proechimys*, interstitial telomeric sequences (ITSs) have not been found in any of the *Proechimys* analyzed up to now. Telomeric signals have been observed only in the distal portions of the chromosomes. The lack of ITSs in this genus, in spite of its ample variation in diploid numbers, indicates that either *(i)* interstitial telomeric sequences may be present in *Proechimys* species, but have not been detected by FISH, due to the small number of repetitions or the inadequate sensitivity of this analytical technique (Ruiz-Herrera *et al.*, 2008) and/or *(ii)* the interstitial telomeric sequences have been eroded or substituted by heterocromatic or satellite DNA sequences following rearrangements, given that they generate fragile sites in the chromosome, and are thus eliminated to avoid breakage.

Given this, while it is still not possible to define precisely the patterns of chromosomal evolution in the genus *Proechimys*, the expansion of both chromosome studies and the number of localities sampled has contributed further to the understanding of the diversity found in this genus. The use of chromosomal painting seems to be a promising way to understand the chromosomal evolution in *Proechimys*, as demonstrated by Oliveira da Silva *et al.* (2019), explaining the origin of the multiple system of sex chromosomes in *P.* cf. *goeldii*, discussing the fixation of chromosomes rearrangements and the sympatric and allopatric models of speciation for the genus. This evidence provides important insights, including new taxonomic markers, and alternative interpretations of the systematics of the group, which emphasize the need for an integrated approach to the understanding of speciation patterns and evolutionary processes, not only in this genus, but in echimyids in general.

## References

[B1] Amaral PJS, Nagamachi CY, Noronha RCR, Costa MJR, Pereira AL, Rossi RV, Mendes-Oliveira ACM, Piczarka JC (2013). Proechimys (Rodentia, Echimyidae): characterization and taxonomic
considerations of a form with a very low diploid number and a multiple sex
chromosome system. BMC Genetics.

[B2] Araújo NP, Loss AC, Cordeiro-Junior DA, da Silva KR, Leite YL, Svartman M (2014). New karyotypes of Atlantic tree rats, genus Phyllomys (Rodentia:
Echimyidae). Genome.

[B3] Bonvicino CR, Otazú IB, Vilela JF (2005). Karyologic and molecular analysis of Proechimys Allen, 1899
(Rodentia, Echimyidae) from the Amazonian region. Arq Mus Nac Rio de Janeiro.

[B4] Cazaux B, Catalan J, Veyrunes F, Douzery EJP, Britton-Davidian J (2011). Are ribosomal DNA clusters rearrangement hotspots? A case study
in the genus Mus (Rodentia, Muridae). BMC Evol Biol.

[B5] da Silva MNF (1998). Four new species of spiny rats of the genus Proechimys (Rodentia:
Echimyidae) from the Western Amazon of Brasil. Proc Biol Soc Wash.

[B6] da Silva MNF, Rylands AB, Patton JL, Capobianco JPR, Veríssimo A, Moreira A (2001). Biogeografia e conservação da mastofauna na floresta amazônica
brasileira. Biodiversidade na Amazônia brasileira: Avaliação e ações prioritárias
para a conservação, uso sustentável e repartição de benefícios. Estação
Liberdade.

[B7] Eler ES, Silva CEF, da Silva MNF, Feldberg E (2012). Comparative cytogenetics of spiny rats of the genus Proechimys
(Rodentia, Echimyidae) from the Amazon region. Genet Mol Res.

[B8] Fabre PH, Galewski T, Tila M, Douzery EJP (2012). Diversification of South American spiny rats (Echimyidae): A
multigene phylogenetic approach. Zool Scr.

[B9] Ford C, Hamerton J (1956). A colchicine, hypothonic citrate, squash sequence for mammalian
chromosomes. Stain Technol.

[B10] Gross MC, Schneider CH, Valente GT, Martins C, Feldberg E (2010). Variability of 18S rDNA locus among Symphysodon fishes:
Chromosomal rearrangements. J Fish Biol.

[B11] Howell WM, Black DA (1980). Controlled silver-staining of nucleolus organizer region with a
protective colloidal developer: A 1-step method. Experientia.

[B12] Iack-Ximenes GE, Vivo M, Percequillo AR (2005). A new genus for Loncheres grandis Wagner, 1845, with taxonomic
comments on other arboreal echimyids (Rodentia, Echimyidae). Arq Mus Nac RJ.

[B13] Ijdo JW, Wells RA, Baldini A, Reeders ST (1991). Improved telomere detection using a telomere repeat probe
(TTAGGG)n generated by PCR. Nucleic Acids Res.

[B14] Kazazian HHJ (2004). Mobile elements: Drivers of genome evolution. Science.

[B15] Machado T, Silva MJJ, Leal-Mesquita ER, Carmignotto AP, Yonenaga-Yassuda Y (2005). Nine karyomorphs for spiny rats of the genus Proechimys
(Echimyidae, Rodentia) from North and Central Brazil. Genet Mol Biol.

[B16] Maia V, Langguth A (1993). Constitutive heterochromatin polymorphism and NORs in Proechimys
cuvieri Petter, 1978 (Rodentia, Echimyidae). Braz J Genet.

[B17] Martins C, Pisano E, Ozouf-Costaz C, Foresti F, Kapoor BG (2007). Chromosomes and repetitive DNAs: A contribution to the knowledge
of fish genome. Fish Cytogenetics.

[B18] Nagamachi CY, Feldberg E, Pieczarka JC, Pereira AL, Silva CEF, Rosa CC, Souza EMS, Pinto JA, Costa MJR, Malcher SM, Mendes-Oliveira AN, Miranda CL (2015). Citogenética de pequenos mamíferos não-voadores da Amazônia
brasileira. Pequenos Mamíferos não-voadores da Amazônia brasileira.

[B19] Oliveira da Silva W, Rodrigues da Costa MJ, Pieczarka JC, Rissino J, Pereira JC, Ferguson-Smith MA, Nagamachi CY (2019). Identification of two independent X-autosome translocations in
closely related mammalian (Proechimys) species. Sci Rep.

[B20] Patton JL (1967). Chromosome studies of certain pocket mice, genus Perognatbus
(Rodentia, Heteromyidae). J Mammal.

[B21] Patton JL, Patterson BD, Timm RM (1987). The species groups of spiny rats, genus Proechimys (Rodentia,
Echimyidae). Studies in Neotropical Mammalogy - Essay in Honor of Philip
Hershkovitz.

[B22] Patton JL, Leite RN, Patton JL, Pardiñas UFJ, D’Elía G (2015). Genus Proechimys. Mammals of South America.

[B23] Patton JL, da Silva MNF, Malcolm JR (2000). Mammals of the rio Juruá and the evolutionary and ecological
diversification of Amazonia. Bull Am Mus Nat Hist.

[B24] Pinkel D, Straume D, Gray JW (1986). Cytogenetic analysis using quantitative, high-sensitivity,
fluorescence hybridization. Proc Natl Acad Sci U S A.

[B25] Reig O, Trainer M, Barros MA (1979). Sur l’ídentification chromosomique de Proechimys guyannensis (E.
Geoffroy, 1803) et de Proechimys cuvieri Petter, 1978 (Rodentia,
Echimyidae). Mammalia.

[B26] Rodrigues da Costa MJ, Amaral PJS, Pieczarka JC, Sampaio MI, Rossi RV, Mendes-Oliveira AC, Noronha RCR, Nagamachi CY (2016). Cryptic species in Proechimys goeldii (Rodentia, Echimyidae)? A
case of molecular and chromosomal differentiation in allopatric
populations. Cytogenet Genome Res.

[B27] Rooney AP, Ward TJ (2005). Evolution of a large ribosomal RNA multigene family in
filamentous fungi: birth and death of a concerted evolution
paradigm. Proc Natl Acad Sci U S A.

[B28] Ruiz-Herrera A, Nergadze SG, Santagostino M, Giulotto E (2008). Telomeric repeats far from the ends: Mechanisms of origin and
role in evolution. Cytogenet Genome Res.

[B29] Silva CEF, Eler ES, da Silva MNF, Feldberg E (2012). Karyological analysis of Proechimys cuvieri and Proechimys
guyannensis (Rodentia, Echimyidae) from central Amazon. Genet Mol Biol.

[B30] Sumner AT (1972). A simple technique for demonstrating centromeric
heterochromatin. Exp Cell Res.

[B31] Vitte C, Fustier MA, Alix K, Tenaillon MI (2014). The bright side of transposons in crop evolution. Brief Funct Genomics.

[B32] Woods CA, Kilpatrick CW, Wilson DE, Reeder DM (2005). Infraorder Hystricognathi. Mammal species of the world: A taxonomic and geographic
reference.

[B33] Yonenaga-Yassuda Y, Souza MJ, Kasahara S, L'abbate M, Chu HT (1985). Supernumerary system in Proechimys iheringi iheringi (Rodentia,
Echimydae) from the state of São Paulo, Brazil. Caryologia.

[B34] Zeigler D, Zeigler D (2014). Transposable elements, viruses, and genomes. Evolution: Components and mechanisms.

